# Increasing aleurone layer number and pericarp yield for elevated nutrient content in maize

**DOI:** 10.1093/g3journal/jkad085

**Published:** 2023-04-18

**Authors:** Michael N Paulsmeyer, John A Juvik

**Affiliations:** Vegetable Crops Research Unit, USDA-ARS, Department of Horticulture, University of Wisconsin at Madison, 1575 Linden Dr., Madison, WI 53706, USA; Department of Crop Sciences, University of Illinois at Urbana-Champaign, 1201 W. Gregory Dr., Urbana, IL 61801, USA

**Keywords:** multilayered aleurone, supernumerary aleurone layer, ICP-OES

## Abstract

The bran is a nutritive fraction of the maize (*Zea mays* L.) kernel containing micronutrients, quality protein, and antioxidants beneficial for human health. Bran consists of two major components: aleurone and pericarp. Increasing this nutritive fraction would therefore have implications on biofortification of maize. Since quantification of these two layers is difficult, the goals of this study were to develop efficient techniques for analyzing these layers and to develop molecular markers for pericarp and aleurone yield. Two populations with various characteristics were genotyped using genotyping-by-sequencing. The first was a yellow corn population with contrasting pericarp thicknesses. The second was a blue corn population segregating for *Intensifier1* alleles. Both populations segregated for the multiple aleurone layer (MAL) trait that is known to increase aleurone yield. In this study, it was found that MALs are mostly determined by a locus on chromosome 8, but several minor loci are also involved. The inheritance of MALs was complex and seemingly more additive than dominant. In the blue corn population, anthocyanin content increased 20 to 30% with the addition of MALs demonstrating its effectiveness at increasing aleurone yield. Elemental analysis was performed on MAL lines and indicated a role of MALs in increasing iron content in the grain. Iron content was increased 17.5% in the MAL lines over the single aleurone layer lines and 35.5% over the recurrent parent, Mo17. Zinc content was increased 15.5% in the MAL lines compared to the recurrent parent. QTL analyses are presented in this study on many pericarp, aleurone, and grain quality traits. Molecular markers were also tested for the MAL locus on chromosome 8, and candidate genes are discussed. Results of this study may assist plant breeders enhancing anthocyanin content and other beneficial phytonutrients in maize.

## Introduction

Breeding plants for increased vitamin, micronutrient, and essential amino acid content is important for ensuring staple crops provide adequate nutrition for human diets. Many resource-scarce societies depend on cereal grains lacking in certain nutrients for a majority of their caloric intake. For example, in Sub-Saharan Africa, maize (*Zea mays* L.) accounts for on average 25% of daily caloric intake with some countries subsisting on 50% of daily caloric intake from maize alone (Nuss and Tanumihardjo 2011). Maize is deficient in essential nutrients iron, zinc, vitamin A, lysine, and tryptophan (Goredema-Matongera *et al*. 2021). Therefore, investigations into methods for increasing the health and nutrition of diets where maize is a staple food source are important.

Pericarp and aleurone are nutritionally beneficial fractions of the maize kernel. During the commercial milling process, pericarp and aleurone are concentrated into what is referred to as the “bran” fraction. This fraction is generally considered a by-product of the milling process, but accumulates many essential micronutrients such as calcium, iron, magnesium, potassium, thiamine, and vitamin E (Luithui *et al*. 2019). In fact, in barley grain, nearly all calcium, magnesium, phosphate, and potassium are within the aleurone alone (Becraft 2007). In addition, maize bran is being considered as a dietary supplement since it contains high proportions of soluble dietary fiber and is enriched in antioxidant ferulic acid (Singh *et al*. 2012; Shin *et al*. 2018). Both pericarp and aleurone layers are independently capable of producing anthocyanins depending on the genetic makeup of the plant. Anthocyanins are also well-known antioxidants and generally recognized as health-promoting compounds (see review He and Giusti 2010). Overall, the bran fraction of cereal grains plays an important role in the nutritional value of the crop.

A conceptually simple approach to enhancing nutrition of maize would be to increase the yield of the bran fraction in the maize kernel. The two layers that comprise the maize bran—pericarp and aleurone—are controlled by different developmental mechanisms. Pericarp is the outermost layer of the kernel consisting of two to twenty cell layers and is maternally-derived tissue (Tracy *et al*. 1978). Numerous studies have investigated pericarp thickness as a quality trait especially for waxy and sweet corn (Ito and Brewbaker 1991; Choe and Rocheford 2012; Wu *et al*. 2020). Increasing pericarp thickness also has implications on plant health as a thick pericarp is associated with increased disease and insect resistance (Hoenisch and Davis 1994; García-Lara *et al.* 2004). Pericarp thickness does not necessarily translate to pericarp yield, so other measures need to be considered. Here, pericarp yield is represented in two ways: proportion of pericarp of total weight or weight of pericarp per kernel.

Aleurone is the epidermal layer of the endosperm and contains many of the components necessary to break the kernel from dormancy and to sustain the seedling during germination. Aleurone is genetically triploid, containing one paternal and two maternal genomes as a result of double fertilization (Becraft and Yi 2011). Typically, aleurone is a single layer of cells around the periphery of the endosperm. However, some tropical landraces are known to produce two to nine cellular layers in what is referred to as the multiple aleurone layer (MAL) trait (Welch *et al*. 1993). The effect of this trait is that it can increase the proportion of aleurone per kernel from around 2% on average to up to 4% in the varieties tested in a previous study (Wolf *et al*. 1972). MALs would be beneficial in increasing anthocyanins in blue corn and also the beneficial phytonutrients stored in this layer. The tedious nature of quantifying pericarp and aleurone yield necessitates the creation of molecular markers associated with increasing these kernel fractions and to better accumulate beneficial phytonutrients contained within those tissues.

## Materials and methods

### Plant materials

Multiple aleurone layer landraces PI 515087 and PI 571553 were provided by the North Central Regional Plant Introduction Station in Ames, IA, USA. Genetic stocks 707G, 805B, 827C, and 827CA were provided by the Maize Genetics Cooperation Stock Center in Urbana, IL, USA. Two populations were developed for the purpose of determining the inheritance of MALs and pericarp yield (Table 1). The first population, designated MAL1, was a yellow corn population from a cross between San Martin 105 (PI 515087) and Mo17. San Martin 105 has the ability to produce five to six aleurone layers and consistently produces MALs in every kernel. These two parents contrasted in the thickness of their pericarps as well. Most MAL landraces have thin pericarps (personal observation), while temperate yellow dent lines tend to have thicker pericarps (Ito and Brewbaker 1991). The second population was a cross between MAL landrace San Martin 119 (PI 571553) and genetic stock 707G from the Maize Genetics Cooperation Stock Center and is designated MAL2. This MAL landrace had the capability to produce three aleurone layers consistently. The genetic stock 707G was chosen because it contains blue aleurone pigmentation along with a recessive *intensifier1* (*in1*) gene, which is known to enhance aleurone pigmentation. Both populations were backcrossed once with their respective non-MAL parent. MAL2 segregated for the blue aleurone trait, so only blue kernel samples were selected after each generation of selfing. MAL1 was phenotyped and genotyped for MALs and pericarp content in the BC_1_ stage and so the aleurone in each kernel is genetically BC_1_F_1_ (Table 1). MAL2 was selfed to the BC_1_F_2:3_ stage, and the families were genotyped and phenotyped (Table 1). To bulk more seed for subsequent years of analysis, plants within families were sib-pollinated to maintain the family structure.

**Table 1.** Populations used in this study.

MAL1			
**Summer 2015**	**San Martin 105 × Mo17**	**Cross**	
**Summer 2016**	**F_1_ × Mo17**	**Backcross**	
**Winter 2017**	**BC_1_ plants, BC_1_F_1_ aleurone**	**Self**	**Phenotype/genotype**
**Summer 2018**	**BC_1_F_1_ plants, BC_1_F_2_ aleurone**	**Self**	
	**BC_2_ × Mo17**	**Backcross**	
**Summer 2019**	**BC_1_F_2_ plants, BC_1_F_2:3_ aleurone**	**Self**	**Phenotype**
	**BC_3_ × Mo17**	**Backcross**	
**Summer 2020**	**BC_4_ × Mo17**	**Backcross**	**Phenotype**
	**Mo17 × BC_4_**	**Backcross**	**Phenotype**
	**BC_3_ × Mo17**	**Backcross**	**Phenotype**
	**Mo17 × BC_3_**	**Backcross**	**Phenotype**
**Summer 2021**	**BC_5_ × Mo17**	**Backcross**	**Phenotype/marker validation**
	**BC_4_ × Mo17**	**Backcross**	**Phenotype/marker validation**

MAL2			
**Summer 2015**	San Martin 119 × 707G	Cross	
**Winter 2016**	F_1_ × 707G	Backcross	
**Summer 2016**	BC_1_ plants, BC_1_F_1_ aleurone	Self	
**Summer 2017**	BC_1_F_1_ plants, BC_1_F_2_ aleurone	Self	
**Summer 2018**	BC_1_F_2_ plants, BC_1_F_2:3_ aleurone	Sib-pollinate	Phenotype/genotype
**Summer 2019**	BC_1_F_2:3_ families	Sib-pollinate	Phenotype

### Phenotypic measurements

**Fig. 1.**
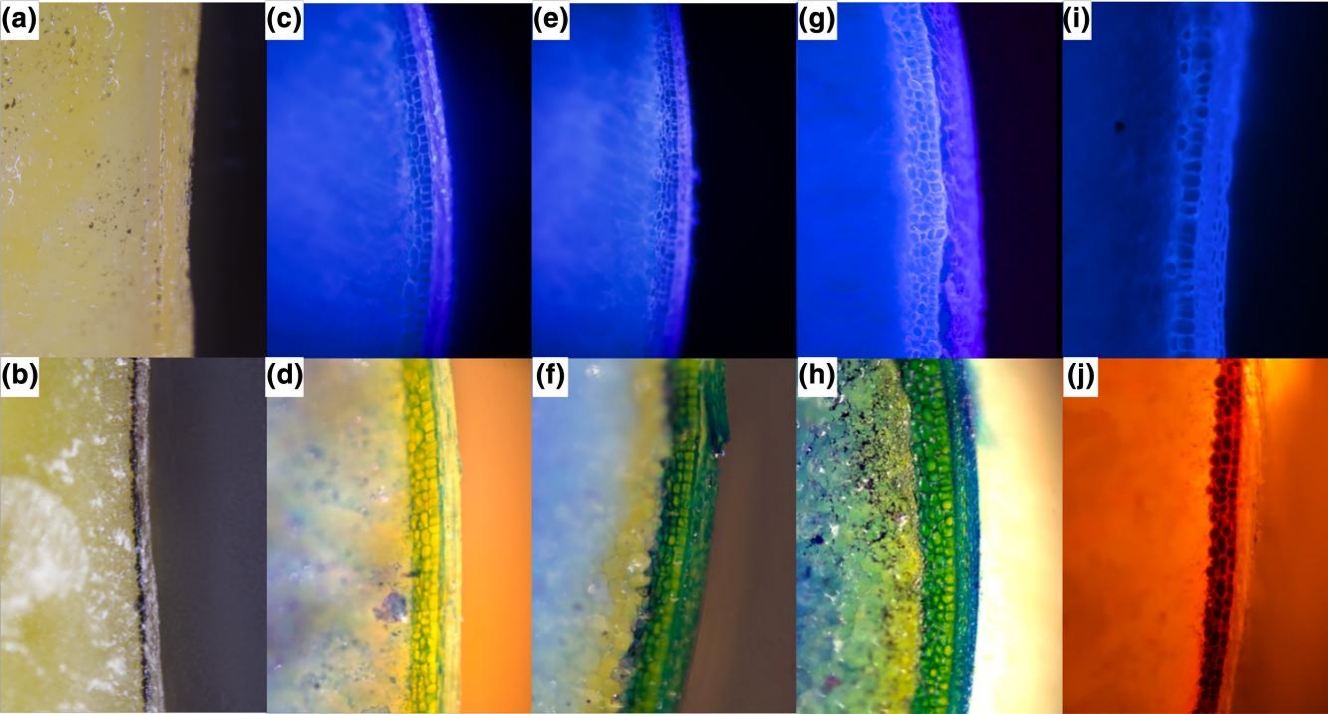
Microscope images of aleurone phenotypes in this study. a and b) Single aleurone layer kernels from Mo17 (a) and 707G (b) viewed with white light. c and d) Kernels with three aleurone layers from San Martin 119 as viewed with the DAPI filter (c) and toluidine blue chemical dye staining (d). e and f) Kernels with three to four aleurone layers from San Martin 119 as viewed with the DAPI filter (e) and toluidine blue staining (f). g and h) Kernels with three to four aleurone layers from the MAL1 yellow corn population as viewed with the DAPI filter (g) and toluidine blue staining (h). i) Kernel with heterogeneous layers as viewed with the DAPI filter. j) Member of the MAL2 blue corn population viewed with the TRITC filter. All images were viewed with a BX-66 Fluorescent microscope (Olympus Corporation) and 10 × magnification.

The number of aleurone layers was assayed by rough sectioning of mature kernels with a razor blade. Kernels were hydrated for at least 16 hours in water at room temperature prior to analysis. Hydrated kernels were cut into 1 to 2 mm sections longitudinally in the center of the kernel for most analyses or sagitally along the crown if the purpose was to germinate the kernel later. The resulting sections were viewed within 3 to 5 min on an Olympus BX-66 Fluorescent microscope (Olympus Corporation) equipped with a triple band DAPI–FITC–TRITC (Tetramethylrhodamine) fluorescence filter and 10 × magnification. Sections could also be stained using 0.1% (*w/v*) toluidine blue for 2 min prior to viewing with white light illumination from above and 10 × magnification (Fig. 1d, f, and h). The DAPI filter allows for viewing of yellow corn and weakly pigmented blue aleurone samples (Fig. 1d, e, g, and i), while the contrast with the red excitation of the TRITC filter contrast blue aleurone cell bodies from aleurone cell walls (Fig. 1j). The number of aleurone layers per kernel was determined as the consensus number of layers as viewed around the entire circumference of the endosperm. These values were averaged among at least five kernels for each MAL1 line and at least ten kernels for MAL2 line. The maximum number of aleurone layers among the kernels assayed was also recorded. Grain quality traits, including moisture, protein, oil, and density were measured for the MAL1 population using the Perten DA 7200 Near Infrared (NIR) Analyzer (Table 2). Two replicates of 10 kernels were weighed and adjusted to 0% moisture using the NIR values. These 10-kernel lots were boiled for 15 min in 20 mM NaOH to soften the kernels and loosen pericarps. Pericarp and kernel fractions were separated using forceps into separate aluminum weigh boats. Fractions were heated at 103°C for 72 hours according to AACC (American Association of Cereal Chemists) approved method 44-40.01 (AACC Approved Methods of Analysis 2009) to remove moisture and weighed immediately. The dry pericarp weight was divided by the moisture-adjusted ten-kernel weight to calculate proportion of pericarp per kernel (Table 2). Average variability of measurements within ear were within 8% CV (standard deviation divided by mean) for proportion of pericarp, so the values are acceptably reproducible. Variability in measurements among ears tended to be higher than within ear measurements (data not shown). A subset of samples (*n* = 19) from MAL1 were selfed twice and analyzed for their aleurone layer number (Table 1). Two 35-gram replicates of non-MAL and MAL samples along with two 35-gram replicates and two ears of Mo17 were run using ICP-OES (inductively coupled plasma-optical emission spectrometry) at Brookside Laboratories Inc. in New Bremen, OH, USA for elemental analysis.

**Table 2.** Summary of phenotypes in the two MAL populations.

Trait			MAL1	MAL2
Prop MAL	%		74.40	60.20
AVG	Avg		1.36	1.20
	*H* ^2^		—	0.68
ADJ	Avg		1.49	1.36
	*H* ^2^		—	0.86
ACN			Homozygous recessive *intensifier1 (in1)*	
	Avg (mg kg^−1^)		Single	178.54
			Multiple	238.54
			Homozygous dominant *Intensifier 1 (In1)*	
	Avg (mg kg^−1^)		Single	122.61
			Multiple	90.32
	*H* ^2^		—	0.95
Protein	Range %		8.45–13.82	—
	Avg %		11.51	—
Oil	Range %		2.51–5.29	—
	Avg %		3.77	—
KD	Range (g cm^−3^)		1.21–1.36	—
	Avg (g cm^−3^)		1.29	—
KV	Range (cm^3^)			—
	Avg (cm^3^)		2.69	—
KWT	Range (mg)		140.49–469.49	—
	Avg (mg)		348.62	—
PCT	Range %		2.99–7.22	—
	Avg %		4.16	—
PWT	Range (mg)		4.63–18.78	—
	Avg (mg)		12.93	—
Fiber	Range %	0.71–2.46	—	
	Avg %	1.50	—	

ACN, anthocyanin content (mg anthocyanin per kg maize powder); ADJ, adjusted number of aleurone layers based on maximum layers possible in individual kernels; AVG, average number of aleurone layers; KD, kernel density (g cm^−3^); KV, kernel volume (cm^3^); KWT, kernel weight per kernel (mg); PCT, proportion of pericarp per kernel (%); Prop MAL, proportion of individuals containing the MAL phenotype; PWT, pericarp weight per kernel (mg).

### GBS library construction

Genomic DNA for this study was extracted using a CTAB procedure modified for 96-well plates (Doyle and Doyle 1990). A double restriction enzyme genotyping-by sequencing approach was used to generate genetic markers associated with MAL formation (Poland *et al*. 2012). The restriction enzymes used to generate the libraries were *Hin*P1I and *Pst*I. The total number of segregating lines sampled in MAL1 and MAL2 was 165 and 150, respectively. Additional samples from external experiments were included within the libraries to bring the total to 192 samples in each library. Libraries were sequenced at the DNA Services laboratory of the Roy J. Carver Biotechnology Center at the University of Illinois at Urbana-Champaign. Before sequencing, libraries were cleaned one additional time using a 1:1 ratio with AxyPrep Mag PCR Cleanup beads (Axygen, Inc.) to ensure removal of primer and adaptor dimers, then quantitated on Qubit (Life Technologies) and evaluated on AATI Fragment Analyzer (Advanced Analytics). The pools were diluted to 5 nM concentration and further quantified by qPCR on a BioRad CFX Connect Real-Time System (Bio-Rad Laboratories, Inc.). The two final pools were loaded onto separate lanes and then sequenced on an Illumina HiSeq4000 with version 1 SBS sequencing reagents from one end of the molecules for a total read length of 100 nt. Raw data was adapter-trimmed and converted to fastq format using bcl2fastq v2.20 Conversion Software (Illumina). Four samples were removed from MAL2 after it was found that they were a result of pollination contamination. This brings the total number of samples to 165 for MAL1 and 146 for MAL2.

### SNP discovery pipeline

Raw reads were de-multiplexed, and the barcodes were removed using Stacks 2.54 (Catchen *et al*. 2013). Reads with a quality score of less than 30 were removed. Taxa were aligned using BWA-MEM using the Mo17 genome for MAL1 and the B73 RefGen_v4 genome for MAL2 (Li and Durbin 2010; Jiao *et al*. 2017; Sun *et al*. 2018). SNP discovery used the SAMtools 1.11 and BCFtools 1.9 pipelines (Bonfield *et al*. 2021; Danecek *et al*. 2021). Genotypes were called using the multiallelic calling model (Danecek *et al*. 2014). SNPs with a read depth greater than 6 and a minimum quality score of 30 were kept for analysis. Taxa with less than 100,000 reads were removed after this step, which removed one sample from MAL1 and two samples from MAL2. The total number of samples for the final dataset was 164 for MAL1 and 144 for MAL2. This dataset was then fed into TASSEL 5.0 where the dataset was filtered to remove missing information and any sites that had a minor allele frequency less than 0.05 and heterozygosity frequency greater than 0.9 (Bradbury *et al*. 2007). The cleaned dataset was thinned so that markers were spaced at least 10,000 base pairs apart since closely linked markers do not add additional information. This resulted in 3871 SNPs for MAL1 and 5403 SNPs for MAL2. GBS (genotype by sequencing) data is filled with errors in heterozygote calling due to low-depth sequencing. In addition, the maize genome is repetitive sequences and duplications that make alignment difficult (Jiao *et al*. 2017). A custom script was developed in R to smooth the dataset and correct the errors similar to the bin-mapping method from a previous study analyzing GBS data (Chen *et al*. 2014; R Core Team 2019). MAL1 was smoothed with a window of 20 SNPs with a step size of 2 SNPs. MAL2 was smoothed with a window of 40 SNPs with a step size of 2 SNPs. If a window was 75% homozygous for either the reference or alternate allele, then the middle position of that window was reported as homozygous. If not, then it was considered heterozygous. This resulted in 1835 high quality SNPs in MAL1 and 2498 high quality SNPs in MAL2.

### Molecular marker analysis

Several PCR-based markers were used in this study. Many of these markers were publicly available from the Maize Genetics and Genomics Database (https://maizegdb.org/data_center/ssr) (Andorf *et al*. 2010). Others were custom designed based on polymorphic sequence content between genomic sequences of B73 and Mo17 (Jiao *et al*. 2017; Sun *et al*. 2018). Eight markers chosen were spaced across an interval on chromosome 8 from 19.1 Mb to 27.1 Mb in Mo17 (Supplementary Table 1). Most markers were validated in the MAL1 population and are known to be polymorphic. *MPMAL1*, *MPMAL2*, and *MPMAL3* custom markers fully aligned to each maize genome available on the Maize Genomics and Genetics Database (Cannon *et al*. 2011). The reaction consisted of 1.2 M betaine, 600 nm of each primer, and 1 to 100 ng DNA in Taq 5X Master Mix (New England Biolabs). The thermal cycler program was the same for each marker except for the annealing temperature listed in Supplementary Table 1. The cycles used were 1 min initial denaturation at 94°C followed by 35 cycles of 30 seconds at 94°C, 30 seconds at the annealing temperature, and 30 seconds at 72°C; and ending with 3 min at 72°C. Additionally, custom RFLP marker *MPMAL4* was designed based on GBS reads (Supplementary Table 1). A restriction enzyme assay consisted of 20% (*v*/*v*) PCR product, 1X CutSmart Buffer (New England Biolabs), and 0.5 units *Eae*I run for 1 hour at 37°C. The PCR products were separated on a 4% SFR agarose gel (VWR) while restriction enzyme products were separated on a 2% gel in ice-cold TBE at 3.9 V cm^−1^. Running the gel for 1.5 to 2.5 hours was sufficient for all SSR markers, while 45 min was sufficient to separate the restriction enzyme marker.

### Statistical analyses and QTL mapping

Elemental analysis data was averaged among the replicates and elemental composition between the single vs multiple aleurone layer lines was compared using the Tukey's honest significant difference method in R (R Core Team 2019). Phenotypes for MAL2 were a result of multiyear trial, so variance components and Best Linear Unbiased Predictions could be calculated. Mixed models were run using the *lme4* package with defaults and the model in Equation 1 (Bates *et al*. 2015). In this equation, term *y* is the phenotypic values, μ is the grand mean, α is the random effect of year, β is the random effect of year, γ is the random effect of genotype, αγ is the random year × genotype interaction term, and ε is the residual.


(1) $$y_{\mathrm{ijkl}}=\mu +\alpha_{i}+\beta_{j(i)}+\gamma_{k}+\alpha \gamma_{\mathrm{ik}}+\varepsilon_{(\mathrm{ijk})l}$$

Estimated variance components were used in Equation 2 to calculate broad-sense heritability (*H^2^*). In Equation 2, *e* is the number of years (*e* = 2) and *r* is the average number of replications per year (*r* = 1.5).


(2) $$H^{2}=\frac{\sigma_{g}^{2}}{\sigma_{g}^{2}+\frac{\sigma_{g\times e}^{2}}{e}+\frac{\sigma_{e}^{2}}{r*e}}$$

A genetic map was estimated using the Carter–Falconer method in the “qtl” package in R (Carter and Falconer 1951; Broman *et al*. 2003). Conditional genotype probabilities were calculated with fixed step width of 0.1 using “calc.genoprob”. QTL mapping was performed using the “Expectation–Maximization” method in “scanone” and the 1.5-LOD interval was calculated using “lodinterval”. Thresholds for each trait were calculated using 1000 permutations (Broman *et al*. 2003). The most significant markers that were significantly above the LOD threshold were run in a linear model in “fitqtl” to calculate “Type III” sums of squares, additive and dominance estimates, and the partial *R*^2^ values (Broman *et al*. 2003). Estimated effects of each marker were calculated using a linear model in R (R Core Team 2019).

## Results

### Multiple loci confer the MAL trait

The number of loci conferring MALs differed depending on the MAL landrace used for the two populations. MAL1 was phenotyped and genotyped at the BC_1_ generation and so frequencies of MAL are expected to be 50% for a single locus or 75% for two loci assuming redundant dominant loci with no epistasis. MAL2 was phenotyped and genotyped at the BC_1_F_2:3_ stage and so the frequency of MAL individuals is expected to be 28.1% for a single locus system, 48.3% for a two-locus system, and 60.2% for a three-locus system, also assuming no epistasis and redundant dominant loci. The null hypothesis that two loci are involved with the MAL trait in MAL1 cannot be rejected given the ratio of MAL individuals in Table 2 (χ^2^*P* = 0.87). The number of MAL individuals in the MAL2 population follows the expected ratio for three loci (χ^2^*P* = 1).

Families from MAL2 were replicated across two years to calculate broad-sense heritability (*H^2^*). The MAL trait is moderately heritable in this population with a relatively large contribution of genotype × environment interactions (Table 2). MAL counts were determined based on the consensus number of aleurone layers. Often, a kernel would have a heterogeneous distribution of aleurone layers across the crown to the tip (Fig. 1). In these cases, the maximum layers possible were also recorded. Adding a correction for this value resulted in a large increase in genotypic variance and an increase in *H*^2^ from 0.68 to 0.86 (Table 2). The MAL trait has a strong genetic component but can be influenced by the environment or other unknown factors to produce more or fewer aleurone layers.

### Reciprocal crosses indicate that the MAL trait is mainly additive

To determine if there was a dosage effect with MAL alleles, reciprocal crosses were made between single layer and MAL lines. Aleurone is triploid, receiving one paternal and two maternal genomes, so it is expected there will be a parental effect if the inheritance is more additive. To reduce genetic variability, members from MAL1 were backcrossed two or three additional times to Mo17, the recurrent temperate yellow dent inbred (Table 1). In the reciprocal cross study, ears were designated females if they received pollen from the single aleurone parent or males if they donated pollen to the single aleurone parent. Several female ears with up to four aleurone layers, and MALs in at least 75% of kernels were found indicating that at least two MAL genes were still segregating even after three backcrosses in this population. These four layer lines were grown the following summer and reciprocal crossed to Mo17 once again to test the inheritance of MAL formation. An average of 47% of female kernels (*n* = 240) and 16% of the male MAL kernels produced MALs (*n* = 77). These testcrosses had significantly fewer MALs than expected for a two dominant gene system (χ^2^*P* < 0.0001). The average number of layers per ear was 1.62 for female ears (*n* = 9) and 1.13 for male ears (*n* = 7). This was statistically significantly different in a paired *t*-test (*P* = 0.016). The reciprocal cross experiment determines that MAL formation can be additive, especially in the MAL1 population analyzed, and that increasing the dosage of MAL alleles increases aleurone layer number.

### Pericarp yield

Many studies have investigated pericarp thickness, but this trait does not necessarily translate to pericarp yield on a per-kernel or per-weight basis. Two separate measurements were made to calculate pericarp yield here: pericarp yield in terms of weight per kernel and pericarp yield in terms of proportion of total weight. MAL landrace San Martin 105 had an average of 14.18 mg pericarp per kernel, which was a proportion of 4.97% by weight. Mo17 had an average of 7.87 mg pericarp per kernel, or 5.56%. Proportion of pericarp by weight was not significantly different between the two parents with three replicates. The kernels were larger for San Martin 105 than Mo17 when they were sampled, which may explain the higher pericarp yield per kernel. A larger kernel volume (calculated as 10-kernel weight divided by density) and kernel weight was highly correlated with pericarp weight per kernel, but density was only loosely correlated with these traits (Table 3). In this study, pericarp weight per kernel and proportion of pericarp by weight were normally distributed despite only one round of recombination through backcrossing before measurements. The amount of variability was relatively high considering the parents differed only slightly in their proportion of pericarp by weight. There was a range of 4.63 to 18.78 mg pericarp per kernel and 2.99 to 7.22% pericarp by weight in MAL1 (Table 2). Pericarp volume and pericarp weight were moderately positively correlated in this population (Table 3), which suggests that these traits could be controlled by different factors that can be improved simultaneously. Fiber content and oil content were negatively correlated with kernel weight and pericarp weight. Protein had a positive association with pericarp yield and kernel weight.

**Table 3.** Correlation matrix of phenotypes in MAL1.

	AVG	ADJ	KWT	PWT	PCT	Protein	Oil	KD	KV	Fiber
MAX	0.89***	0.83***	−0.01	0.01	0.03	−0.04	0.13	−0.04	0	0
AVG		0.93***	−0.02	−0.04	−0.01	−0.09	0.24**	−0.12	0	0
ADJ			−0.04	−0.06	0	−0.08	0.21**	−0.10	−0.03	0
KWT				0.79***	−0.27***	0.18*	−0.32***	0.27***	0.99***	−0.21**
PWT					0.35***	0.33***	−0.38***	0.22**	0.78***	−0.17*
PCT						0.26***	−0.08	−0.09	−0.27***	0.08
Protein							−0.32***	0.27***	0.14	−0.26***
Oil								−0.67***	−0.22**	0.60***
KD									0.12	−0.99***
KV										−0.06

Asterisks denote significance where (***) < 0.001, (**) < 0.01, and (*) < 0.05.

ADJ, adjusted number of aleurone layers based on maximum layers possible in individual kernels; AVG, average number of aleurone layers; KD, kernel density; KV, kernel volume; KWT, kernel weight per kernel; MAX, maximum aleurone layers per taxa; PCT, proportion of pericarp per kernel; PWT, pericarp weight per kernel.

### Investigating the effect of MALs on nutritional quality of maize

The distribution of anthocyanin content was complex. The data appears to be skewed towards lower values (less than 100 mg/kg) indicating that *In1* was segregating. The highest values belonged to those families containing MALs with recessive *in1.* Estimated anthocyanin content and the log-transformation of anthocyanin content were both significantly positively correlated with the average number of MALs (ρ = 0.458 and 0.384, respectively). As the maximum aleurone layer number increased, so did anthocyanin content. Overall, there was a 20 to 30% increase in estimated anthocyanin production just due to the presence of MALs in dominant and recessive *In1* lines, respectively (Table 2). An ANOVA determined that both MALs (*P* = 0.00143) and *In1* (*P* = 2.14e^−08^) were significantly increasing estimated anthocyanin content (adjusted *R*^2^ = 0.434). The highest anthocyanin content in MAL2 was in a family with *in1* and a maximum of three layers. The predicted anthocyanin content over the two years studied was 457 mg anthocyanins per kg kernel weight. Using a mixed linear model, anthocyanin content was also heavily controlled by genetic factors in this population with nearly all variation due to genetics.

Most other quality traits did not increase with the presence of MALs. There was a small but significant increase in oil content in MAL1 (Table 3). To assess the micronutrient content, elemental analysis with ICP-OES of single aleurone layer and MAL lines was conducted. The population chosen for study was the MAL1 yellow corn population. Individual selfed ears from the BC_1_F_2:3_ generation were assessed for their aleurone layer content (Table 1). Eight single aleurone layer and eleven MAL ears were represented. Data is shown in Table 4. Iron content was significantly increased in MAL lines, indicating a role of MALs in increasing micronutrient content. Iron content was increased 17.5% in the MAL lines over the single aleurone layer lines and 35.5% over the recurrent parent, Mo17. Zinc content was increased 15.5% in the MAL lines compared to the recurrent parent.

**Table 4.** ICP-OES analysis of non-MAL vs MAL kernels.

		Counts	P	K	Mg	S	B	Fe	Mn	Cu	Zn	Al	Protein	Oil
		No.	pct	pct	pct	pct	ppm	ppm	ppm	ppm	ppm	ppm	%	%
Single	Avg	1.000	0.2945	0.3625	0.1101	0.1133	2.5000	25.2375^a^	7.1375	0.9313	22.5125	6.7625	11.1088	3.8100
	SD		0.0175	0.0141	0.0077	0.0049	0.4899	5.5689	0.7984	0.5339	1.2550	3.8181	0.8413	0.8581
Multiple	Avg	2.3545	0.3368	0.0105	0.1046	0.1071	2.3045	29.4046^a^	6.8318	1.1682	23.0682	7.3450	11.3260	4.0360
	SD		0.0444	0.0400	0.0021	0.0117	0.0138	0.4990	5.8825	1.7951	0.6938	3.6196	1.3770	0.904
Mo17	Avg	1	0.2810	0.3175	0.09875	0.10875	2.075	21.925	6.1250	1.075	20.0000	6.6000	11.7950	3.4900
	SD		0.0134	0.0177	0.0060	0.0004	0.1768	1.0960	0.3182	0.1061	0.9192	0.4243	1.2990	0.3464

Denotes a significant difference between single and multiple aleurone samples according to the Tukey's honest significant difference test (*P* < 0.05).

### QTL mapping in the MAL1 population

**Fig. 2.**
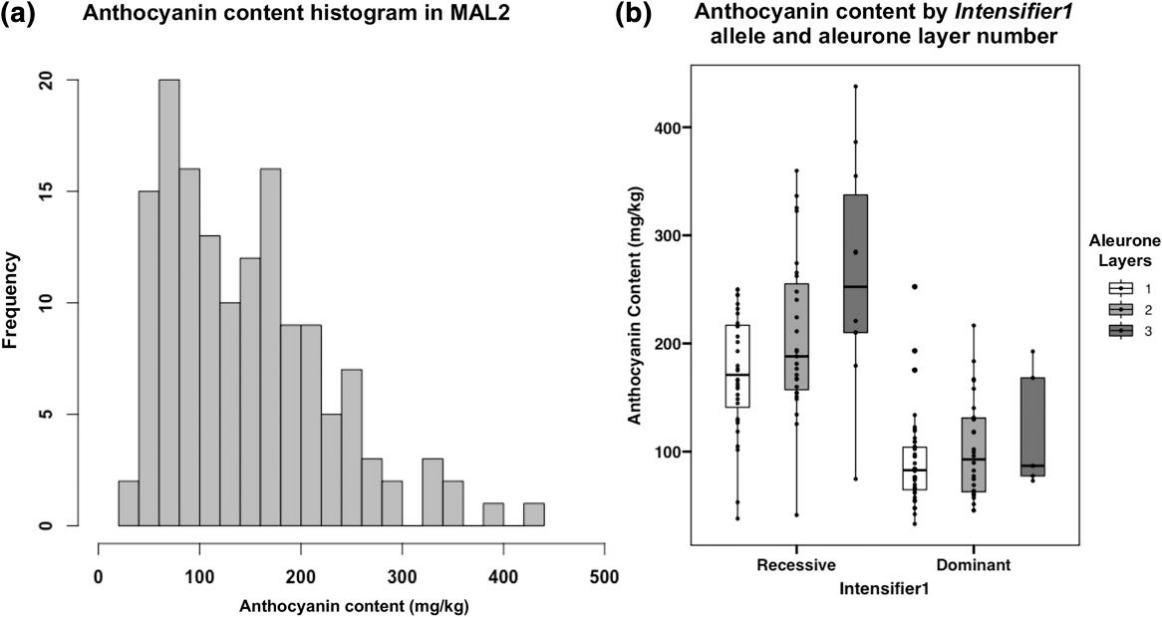
Anthocyanin content increases with the number of aleurone layers. a) Histogram of anthocyanin concentration among families in MAL2 in terms of mg anthocyanins per kg weight of kernel. b) The effect of *Intensifier1* alleles and aleurone layer number on anthocyanin content among families in MAL2. Aleurone layers refer to the maximum number of layers possible in each family.

A major effect QTL on chromosome 8 was found for increased number of aleurone layers in this population (Supplementary Fig. 1). This locus encompasses a 1.5-LOD significance interval of 6.9 Mb centered around 21.3 Mb and accounts for 19.39 to 22.14% of the variation in MALs depending on the aleurone measurement (Table 5). It is predicted that there are at least two loci conferring MALs in this population based on the segregation pattern. Many MAL measurements only had a single significant locus, but average aleurone layers had an additional QTL on chromosome 1 (Supplementary Fig. 1). This SNP marker was located around 219.7 Mb and accounted for 5.98% of the total variation in average aleurone layers. As indicated above, it appears that the MAL trait is additive and not epistatic with the minor locus. Having MAL alleles from both QTL increased the average aleurone layer number more than just having one of each allele (Fig. 2).

**Table 5.** QTL analysis in the MAL1 population.

Trait	Chr	Pos	1.5-LOD CI (Mb)	Interval (cM)	LOD	% *R*^2^
ADJ	8	21,306,878	18.78–25.67	28.2–37.9	7.86	19.39
AVG	1	219,711,643	207.5–251.94	122.7–151.1	2.97	5.98
	8	21,306,878	18.78–25.67	28.1–37.9	9.97	22.14
KD	2	40,455,703	40.46–46.26	50.2–53.8	17.24	26.13
	3	230,689,895	225.26–232.45	126.4–145.6	5.23	6.70
	4	165,684,575	15.02–185.78	45.5–88.7	4.27	5.40
	5	24,463,369	9.68–166.45	36.5–78.7	5.04	6.43
KV	6	5,669,131	5.67–29.08	0–9.7	4.67	11.75
KWT	5	68,327,847	7.62–126.07	31.8–71.4	3.74	8.41
	6	5,669,131	5.67–30.05	0–10.1	5.04	11.55
MAX	8	19,128,271	16.45–24.02	26–36	9.07	22.01
Oil	2	43,972,433	35.17–66.4	46.6–62.5	3.18	6.07
	4	161,861,261	34.53–168.96	59.9–77.6	3.59	6.87
	8	125,739,791	19.13–142.06	29.8–65.7	4.19	8.09
	9	23,085,929	13.41–142.49	21.6–68.7	2.48	4.68
PCT	5	88,876,985	52.14–157.65	59.3–70.1	5.74	14.25
PWT	6	6,443,052	6.44–9.01	0–3.6	9.91	23.30
FIBER	2	41,851,329	40.46–41.85	50–1–53.8	20.03	29.91
	3	230,483,814	218.97–233.51	111.7–147.0	5.46	7.23
	4	165,684,575	13.04–185.78	40.2–89.6	3.64	4.50

ADJ, adjusted number of aleurone layers based on maximum layers possible in individual kernels; AVG, average number of aleurone layers; KD, kernel density; KV, kernel volume; KWT, kernel weight; MAX, maximum aleurone layers per taxa; PCT, proportion of pericarp per kernel (%); PWT, pericarp weight per kernel.

Pericarp traits volume and weight were controlled by different loci, which is logical since they were only moderately correlated (Table 3). Kernel volume, pericarp weight per kernel, and kernel weight all had a significant locus at the start of chromosome 6 (Table 5). The proportion of pericarp by weight and kernel weight had a QTL on chromosome 5 around 88.9 Mb that accounted for 8.42 to 14.25% of the variation in proportion of pericarp (Table 5).

Additional gain quality traits assessed by NIR spectroscopy were also mapped and included in Table 5. It appears that a QTL for oil content overlaps with the MAL trait, although the most significant marker is distal to the MAL markers. In addition, there were significant interactions between oil content, fiber content, and density. Oil content and fiber content were highly negatively associated with density. These traits shared a major effect QTL on chromosome 2 and a minor effect QTL on chromosome 4 around 161.8 Mb that should be investigated further.

### QTL mapping in the MAL2 population

The MAL2 blue corn population was theoretically segregating for at least three MAL loci according to the proportion of taxa with MALs (Table 2). The highest effect locus for MAL formation was due to a chromosome 8 QTL (Table 6). In most cases, this was the only significant QTL (Supplementary Fig. 2). This chromosome 8 QTL overlaps with the most significant MAL1 QTL, but the 1.5-LOD confidence interval placed the MAL2 chromosome 8 QTL much further downstream of the MAL1 QTL even when compensating for differences in genome size. When considering the maximum number of layers possible in the kernel, two additional loci were significantly greater than the LOD threshold (Supplementary Fig. 2). These loci contributed 0.21 to 1.17% of the variation in adjusted aleurone layers, which was not significant in a linear model (Table 6). It may be that these loci are epistatic or affected by epigenetic factors, but a larger population size is needed to calculate a difference. The major chromosome 8 locus in this population acted in an additive manner just as the two MAL loci in MAL1. The ratio of additive to dominance deviations for MAL alleles is shown in Table 6, and the effect of the QTL can be visualized in Fig. 2.

**Table 6.** QTL analysis in the MAL2 population.

Trait	Chr	Pos	1.5-LOD CI (Mb)	Interval (cM)	Add	Dom	LOD	% *R*^2^
ACN	2	219,323,824	211.87–236.99	101.8–118.4	0.188	−0.070	2.16	3.06
	3	187,782,813	175.09–190.2	31.6–37.8	−0.071	0.016	0.38	0.53
	7	11,678,763	9.28–31.63	6.8–11.2	−0.370	−0.155	8.63	13.55
	8	72,353,093	41.89–102.92	6.7–19.9	0.214	0.163	6.70	10.20
ADJ	6	174,199,500	174.09–176.09	65–71.3	−0.043	0.002	0.19	0.21
	8	27,568,159	27.57–28.93	2.5–4.9	0.468	−0.015	23.87	39.47
	9	156,275,580	155.84–157.81	42.7–48.4	−0.030	0.052	1.03	1.17
AFT	2	24,208,985	22.4–40.9	30.7–56.1	0.007	−0.126	6.40	15.25
	8	152,203,849	149.94–167.21	39.2–42.3	−0.070	0.149	5.11	11.90
AVG	8	27,568,159	27.57–28.93	2.8–5	0.302	−0.085	28.59	59.41
LogACN	2	219,323,824	212.88–226.46	101.9–108	0.188	−0.071	2.03	2.78
	3	187,782,813	180.82–190.2	33–37.7	−0.071	0.016	0.30	0.41
	7	11,678,763	9.28–32.34	6.9–11.4	−0.370	−0.155	9.87	15.33
	8	72,353,093	69.36–79.33	12–14.9	0.215	0.162	6.11	8.93
Max	8	28,934,979	27.57–28.93	2.4–22.9	1.085	−0.159	23.97	53.05

ACN, anthocyanin content (mg anthocyanin per kg maize powder); ADJ, adjusted number of aleurone layers based on maximum layers possible in individual kernels; AFT, average flowering time; AVG, average number of aleurone layers; LogACN, natural log transformation of ACN; MAX, maximum aleurone layers per taxa.

**Fig. 3.**
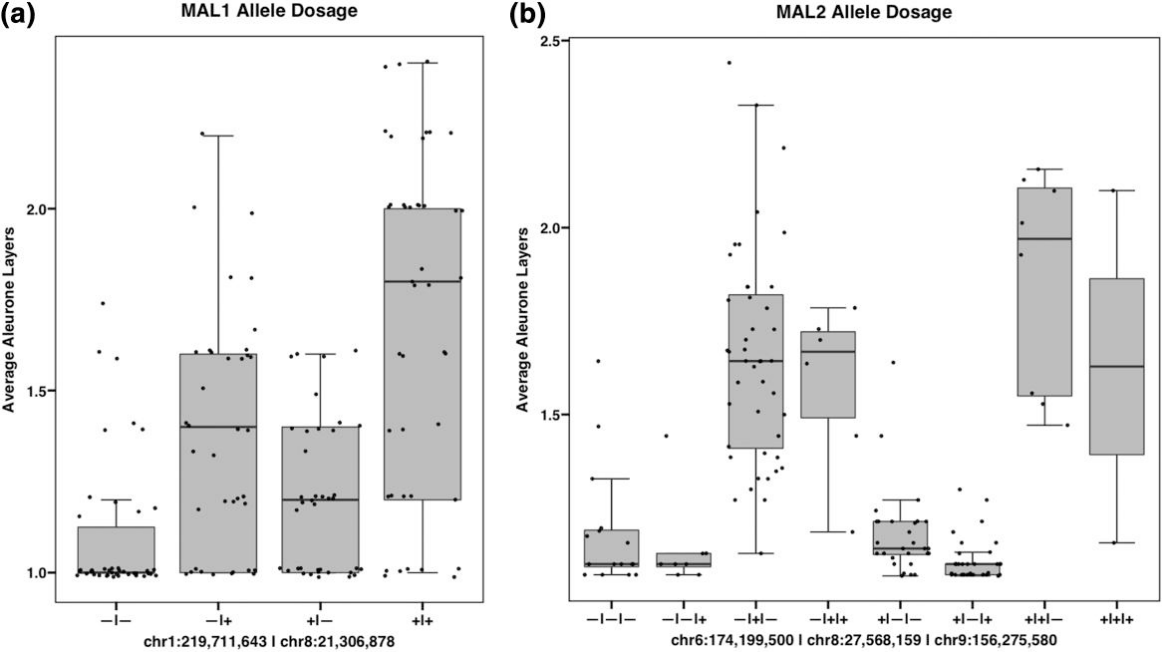
Dosage of significant MAL QTL in the MAL1 and MAL2 populations. Shown are boxplots for the average number of aleurone layers per taxa separated by alleles at each significant QTL. Genotypes are represented as +/− for presence or absence of the MAL QTL. a) Genotypes in MAL1 are for markers chr1:219,711,643 | chr8:21,306,878. b) Genotypes in MAL2 are for markers chr6:174,199,500 | chr8:27,568,159 | chr9:156,275,580.

Anthocyanin content was measured across two years. The distribution of predicted anthocyanin content was skewed (Fig. 3), so the log-transformation of anthocyanin content was mapped. Four loci could be detected (Supplementary Fig. 2). The highest effect QTL contained the gene *in1*, which is known to have a large effect on anthocyanin content (Paulsmeyer *et al*. 2017). The second highest effect locus was on the same chromosome arm as the QTL for MAL formation, although the 1.5-LOD confidence interval was much distal to the MAL locus. There may be linked loci on chromosome 8 that confer higher anthocyanin content, but a larger population would be needed to break linkages. Anthocyanin content increased significantly as aleurone layers increased, so this finding demonstrates that anthocyanin content can be increased with the inclusion of this major effect locus. The other two loci were relatively minor but assisted in increasing anthocyanin content (Table 6). These did not overlap with any canonical anthocyanin biosynthetic or regulatory genes, which is logical since unpigmented kernels were culled from the population.

### Validation of molecular markers linked to MAL formation

Several PCR-based markers were developed within the chromosome 8 MAL QTL, and others were freely available from the Maize Genetics and Genomics Database (Andorf *et al*. 2010). MAL1 was backcrossed to Mo17 four or five times to create heterozygous near-isogenic MAL lines. Many of these were capable of producing up to three layers despite being heterozygous. In the near-isogenic lines, SSR marker umc1530 is 3.12 Mb from the most significant chromosome 8 QTL in this population and was unlinked from MAL formation in the near-isogenic lines (χ^2^*P* = 0.63 for 1:1 MAL:single). MPMAL1, MPMAL2, and umc1913 are 4.3, 628.2, and 239.8 kb away from the most significant chromosome 8 QTL marker from GBS data in the MAL1 population, respectively (Supplementary Table 1). These markers were all highly linked to MAL formation (χ^2^*P* < 1e^−14^ for 1:1 MAL:single). MPMAL1 was 3.5 cM away, umc1913 was also 3.5 cM away, and MPMAL2 was 6.1 cM away from the MAL trait. MPMAL1 and umc1913 appear to be good candidate markers for the MAL locus in MAL1. Future work will determine the best markers for the MAL2 population and the applicability of these markers to other populations.

## Discussion

The first report of MALs was from a recurrent-selection study for high-amylose starch utilizing diverse lines (Wolf *et al*. 1969). The first MAL accession was designated “Peru 442” and was later found to be derived from the Coroico landrace, whose region extends from Ecuador into Peru, and to Bolivia and parts of Brazil (Wolf *et al*. 1972). The San Martin landraces used here originate in Peru. The maximum aleurone layers reported for any MAL landrace are nine layers (Duangploy *et al*. 1976). This current study saw a maximum of six layers in the original San Martin 105 parent, but a maximum of four layers in the populations developed. The first published study for MAL inheritance indicated through reciprocal crosses that doubled layers were dominantly inherited (Wolf *et al*. 1969). Other studies agreed that the MAL trait is either dominant or partially dominant (Wolf *et al*. 1972; Nelson and Chang 1974; Duangploy *et al*. 1976; de Miranda 1980). In this study, MAL formation was more additive than dominant, with more allelic doses conferring more aleurone layers on average. Numerous minor loci also affected aleurone layer number, which was in agreement with another study that concluded that at least three genes must be involved (de Miranda 1980). Some of these minor loci, especially in the MAL2 population, contributed very little to total layers. The fact that numerous loci assist in producing MALs indicates that the trait may not be so unique in maize. The reason that modern cultivars do not have MALs is unknown. Through our personal observations, we have never demonstrated deleterious effects on germination or grain yield attributed to MALs. Future work will be aimed at testing performance in near-isogenic lines at a larger scale. We will also more robustly evaluate protein and micronutrient content in these near-isogenic lines to justify breeding efforts for biofortified maize development.

MAL production was a highly heritable trait, especially when considering maximum aleurone layers per ear (Table 2). The improvement in heritability when considering this heterogeneity is most likely due to the parental effect of MAL dosage, since aleurone is triploid. In some cases, such as with the reciprocal cross study, MALs were completely abolished in some kernels. The erasure of MALs may be due to epigenetic effects or other environmental effects not quite understood. Given this, it would be advantageous for maize breeders to introgress MALs into contrasting heterotic pools, so the maximum layers could be combined in the resulting hybrid. Moreover, in a small number of samples, full sets of single aleurone layer alleles seemed to be able to produce MALs. A likely solution to this was recombination between SNP markers and MAL alleles. In addition, the landraces used to develop the population were segregating for the MAL trait. It could be that other minor loci undetected by QTL mapping were conferring MALs in these samples. Larger populations will be needed to parse out the minor loci affecting aleurone layer numbers.

Anthocyanin content exceeded expectations in the MAL2 population (Fig. 3). The highest anthocyanin content family had over two times the concentration of the *in1* parental stock 707G (Paulsmeyer *et al*. 2017). The aleurone layer is only approximately 2% of the kernel by weight (Wolf *et al*. 1972), but was still able to produce an appreciable amount of anthocyanins in MAL2. There was a 20 to 30% increase in anthocyanin content on average with additional aleurone layers (Table 2). This increase in anthocyanin content is similar to the increase in aleurone yield from a previous study investigating MAL effect on aleurone yield (Wolf *et al*. 1972). Aleurone yield measurements were not directly measured in this population, but the stark increase in anthocyanin content is indicative of an overall increase in aleurone tissue yield. Because of the implications of anthocyanins and aleurone tissue with human health, additional health benefits of the MAL trait were also assessed. There was no association with total protein content and MAL formation in MAL1 (Table 3). Additional experiments should be focused on analyzing individual amino acid contents for quality of protein in MAL lines. There was a small significant increase in oil content in MAL lines (Table 3). This increase in oil was interesting since it is known that aleurone accumulates oils for use by the developing seedling (Geisler-Lee and Gallie 2005). Elemental analysis found a significant increase in iron content (Table 4). There was a large range in many of the micronutrients measured, indicating high genetic variability among samples. Environmental variability may have also confounded results. Future work will be aimed at utilizing near-isogenic lines with MALs to remove genetic variability not associated with MALs. The HarvestPlus (https://www.harvestplus.org/) goals for micronutrients in maize for zinc and iron are 38 and 60 μg g^−1^, respectively (Bouis *et al*. 2011). The ranges found in this population did not meet those goals (Table 4). Mo17 appears to be low in micronutrient content, which brought the total micronutrient potential down. Plant breeders should introgress MALs into varieties already improved for micronutrient content to further increase content.

The mechanism for MAL formation in this study is currently unknown, but much work has been done to investigate aleurone layer development in maize. Normal aleurone layer development depends on several factors. First, differentiation depends on positional cues. During endosperm development, the peripheral cells must detect their position in relation to the whole endosperm to differentiate into starchy endosperm or aleurone cells. Defects in this process result in the lack of aleurone, mosaic patches without aleurone, or only aleurone endosperm (Becraft and Asuncion-Crabb 2000; Shen *et al*. 2003). Additionally, proper aleurone layer development is dependent on the plane of cell division. In the later steps of endosperm development, the aleurone must expand to accompany the growing kernel. Cell division during these steps is restricted to either the anticlinal or periclinal plane by a preprophase band of microtubules. This is in contrast with the starchy endosperm cells which are more flexible in their division orientation (Becraft 2007). Aleurone cells entering the starchy endosperm de-differentiate into starchy endosperm through unknown mechanisms. In this study, many of the kernels seem to be defective in this structured division. Generally, the uppermost layer seems to be ordered, cuboidal cells, and the underlying layers are disorganized and globule (Fig. 1). These aleurone cells below the uppermost layer do not de-differentiate into starchy endosperm indicating they also have defects in positional cues. Given there are multiple loci involved, multiple mechanisms may be responsible for MAL formation in our populations.

The largest effect QTL in both populations was on chromosome 8. The 1.5-LOD confidence interval placed MAL2 farther downstream of the MAL1 QTL. It could be that there were two highly linked loci on chromosome 8 contributing to MALs in MAL2, but a larger population would need to be assessed to see a significant difference. Within the 1.5-LOD confidence interval for average aleurone layers, there were 138 genes in MAL1 and 27 genes MAL2, respectively. No major gene candidates could be found in the MAL2 significance interval. Within the chromosome 8 interval of MAL1 is *Gigantea1* (*Gi1*), a circadian clock-associated negative regulator of flowering time in maize (Bendix *et al*. 2013). Many tropical MAL landraces are photoperiod sensitive, and selection pressure for lines adapted to the Midwest could have incidentally caused variation at this locus. To test whether artificial selection was the cause of the chromosome 8 MAL QTL, average pollination dates were recorded for all MAL2 families in 2019. There was a moderate correlation between pollination date and MAL content (ρ = 0.36). No MAL loci overlapped with any QTL for pollination date, however. There was a QTL for pollination date that overlapped with the well-known flowering time locus *Vegetative to generative transition1* (*Vgt1*) on the opposite arm of chromosome 8 from the MAL QTL (Castelletti *et al*. 2014). The recombination frequency between the most significant *Vgt1* marker and the most significant MAL marker in MAL2 is 0.29. The correlation between pollination date and MAL formation could be an artifact of a small population with large linkage blocks.

Another candidate gene for the MAL phenotype on chromosome 8 in MAL1 is *Barren inflorescence1* (*Bif1*). This gene shows defects in organogenesis and results in barren stalks and ears when mutated. The primary mechanism for this gene appears to be defects in auxin transport (Barazesh and McSteen 2008). Treatment with auxin transport inhibitor NPA resulted in MALs in tissue cultures, so it is logical that a mutant with impaired auxin transport like *Bif1* could also result in MALs. Genetic stocks 805B, 805C, and 805CA were assayed for their aleurone layer content. These stocks are testcrosses, so 50% will be *Bif1* dominant. Out of 26 total kernels, none exhibited the MAL phenotype (χ^2^*P* = 0.373), indicating that it is most likely not involved with MAL formation. Other auxin-related genes, *Vanishing tassel2* and auxin-homeostasis protein *clavata3/esr-related26* are also within the chromosome 8 QTL in MAL1 and could theoretically be involved with MAL formation (Phillips *et al*. 2011; Je *et al*. 2016).

An additional goal of this study was to determine markers associated with increased pericarp yield. This study proves that volume of pericarp and weight of pericarp per kernel are controlled by different factors and are only moderately correlated (Tables 3 and 5). Pericarp weight and kernel weight were controlled by a QTL on chromosome 5. This QTL is near a QTL for grain weight and yield using Mo17 as a parent in a previous study (Beavis *et al*. 1994; Veldboom and Lee 1996). The MAL landrace allele decreased pericarp yield at this locus. Kernel volume, pericarp weight per kernel, and kernel weight were controlled by a QTL at the start of chromosome 6 (Supplementary Fig. 1). In contrast to the chromosome 5 QTL, the MAL parent actually increased pericarp yield at this locus (Table 5). No gene candidates could be found for these loci at this time. Developing markers for these significant loci should help assist in breeding. Future work should integrate these loci into other backgrounds to see if they have a similar effect. An unexpected finding of this study was that density of the kernel was neither strongly correlated with kernel mass nor the pericarp traits (Table 3). This low association with pericarp traits allows for the measurement of pericarp traits without kernel hardness confounding the results. The MAL landraces tend to have floury type endosperms, but the gene conferring this endosperm type was unknown. In the QTL analysis, there was a large-effect QTL for density on chromosome 2 where the *Floury endosperm1* gene lies (Table 5) (Holding *et al*. 2007). This locus was also majorly impacting fiber content, which was unexpected. The interplay between density, fiber content, and pericarp weight is complex, which may indicate that pericarp is not the only significant source of fiber in the kernel.

## Conclusions

Increasing the bran fraction of maize grain has never been a direct goal for breeding programs until now. However, it is known that aleurone and pericarp accumulate dietarily important nutrients like calcium, magnesium, phosphate, potassium, and quality protein, while pericarp is a source of dietary fiber (Wolf *et al*. 1972; Becraft 2007; Singh *et al*. 2012; Luithui *et al*. 2019). The implications of increased pericarp and aleurone yield are more far-reaching than anthocyanin content. Biofortification is a tool for breeders that are utilizing now to reduce the incidence of “hidden hunger” or micronutrient deficiencies in staple crops (Bouis *et al*. 2011). The addition of MALs is a promising tool for breeders trying to elevate nutritional content of maize. The MAL trait increased anthocyanin production 20 to 30%, most likely due to an increase in aleurone yield. Iron content was significantly increased in MAL lines, but future studies will have to test micronutrient levels in near-isogenic MAL lines to reduce genetic variability. Breeding for increased pericarp and aleurone yield is difficult because these tissues are difficult to quantify individually. The methods created here to quantify tissue yield have been used to assay thousands of samples efficiently. Molecular markers were validated for MAL formation and similarly could be developed for pericarp yield traits.

## Supplementary Material

jkad085_Supplementary_Data

## Data Availability

Representative MAL stocks generated in this study are available in limited quantity upon request. Data used for QTL mapping is available in supplementary information and also in the project repository at https://github.com/mpaulsmeyer/MAL. Custom code used to generate the genotype data is also available in the project repository. Supplemental material available at G3 online.
