# Supplementary material for: Increasing aleurone layer number and pericarp yield for elevated nutrient content in maize
Source: G3 (Bethesda). 2023 Apr 18;13(7):jkad085. doi: 10.1093/g3journal/jkad085 (PMC10320751; doi:10.1093/g3journal/jkad085)
Supplement: jkad085_Supplementary_Data [file jkad085_supplementary_data.docx]

**Supplementary Figure and Table Legends**

**Figure S1:** Marker-trait associations in the MAL1 yellow corn population

ADJ = adjusted number of aleurone layers based on maximum layers possible in individual kernels, AVG = average number of aleurone layers, KD = kernel density, KV = kernel volume, KWT = kernel weight, MAX = maximum aleurone layers per taxa, PCT = proportion of pericarp per kernel (%), PWT = pericarp weight per kernel.

**Figure S2:** Marker-trait associations in the MAL2 blue corn population

ACN = anthocyanin content, ADJ = adjusted number of aleurone layers based on maximum layers possible in individual kernels. AVG = average number of aleurone layers. FWT = flowering time measured as average deviation from the average. LogACN = natural log transformation of anthocyanin content. MAX = maximum aleurone layers per taxa.

**Table S1:** Primers used in this study

**Figure S1:** Marker-trait associations in the MAL1 yellow corn population

ADJ = adjusted number of aleurone layers based on maximum layers possible in individual kernels, AVG = average number of aleurone layers, KD = kernel density, KV = kernel volume, KWT = kernel weight, MAX = maximum aleurone layers per taxa, PCT = percent pericarp per kernel, PWT = pericarp weight per kernel.


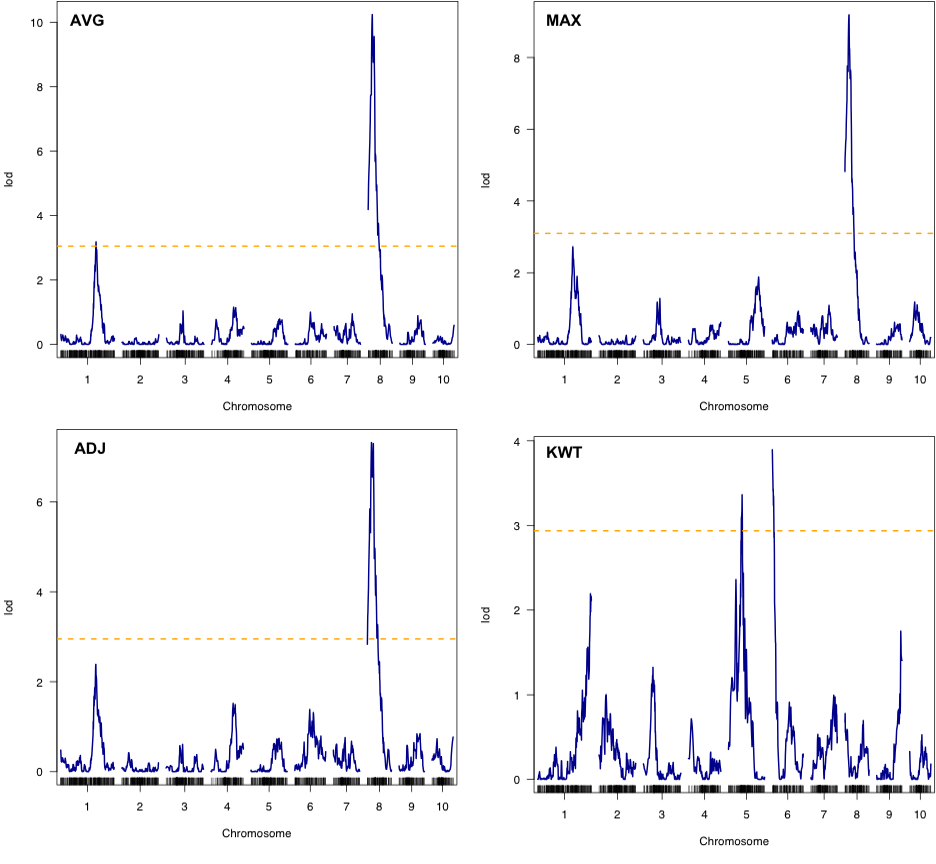


**Figure S1 (Cont.)**


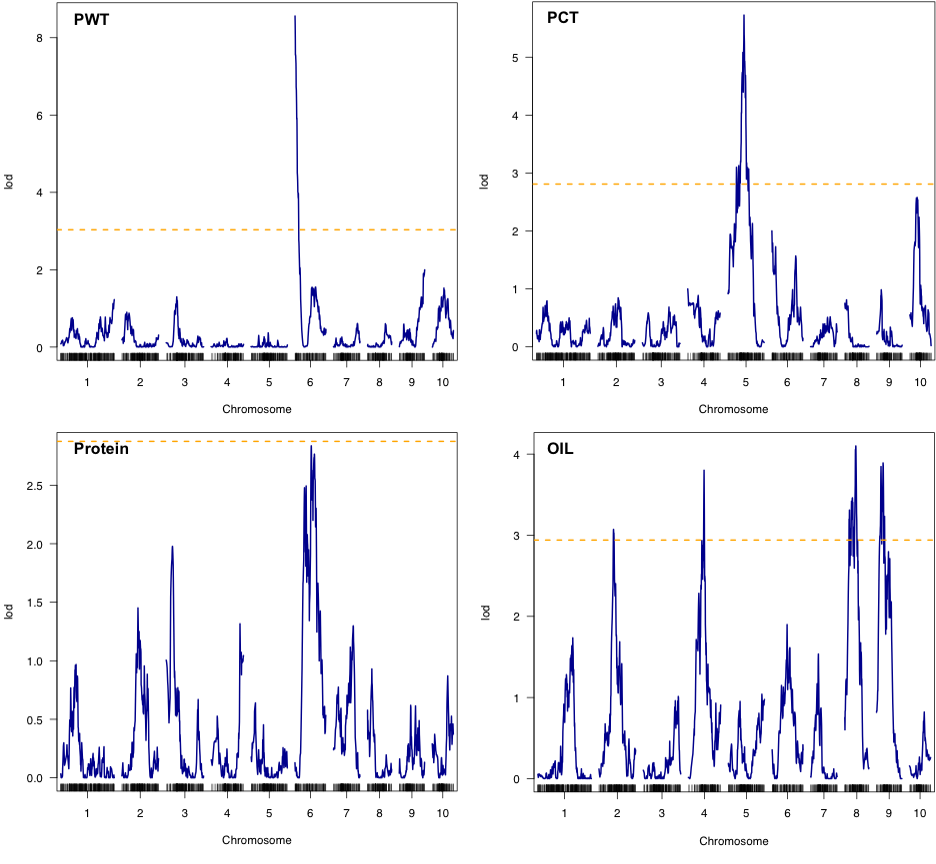


**Figure S1 (Cont.)**


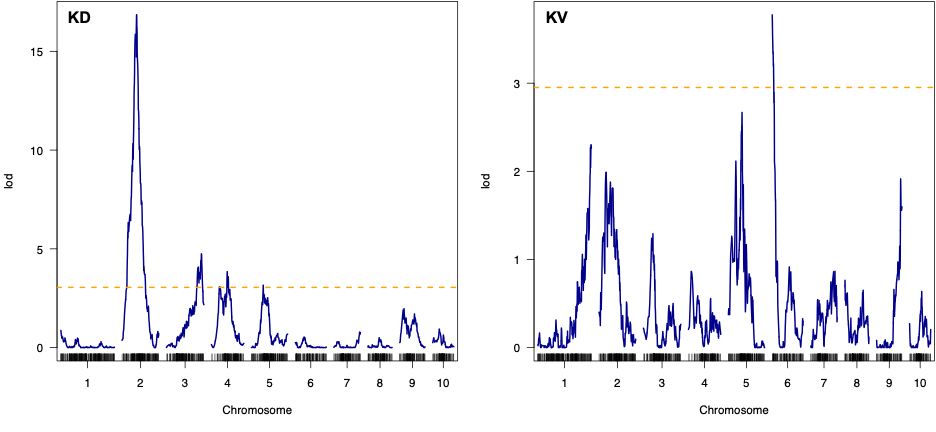


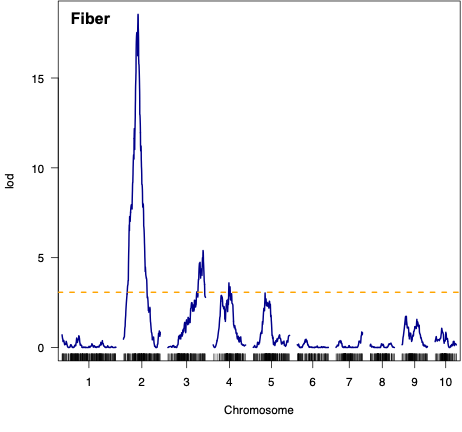


**Figure S2:** Marker-trait associations in the MAL2 blue corn population

ACN = anthocyanin content, ADJ = adjusted number of aleurone layers based on maximum layers possible in individual kernels. AVG = average number of aleurone layers. FWT = flowering time measured as average deviation from the average. LogACN = natural log transformation of anthocyanin content. MAX = maximum aleurone layers per taxa.


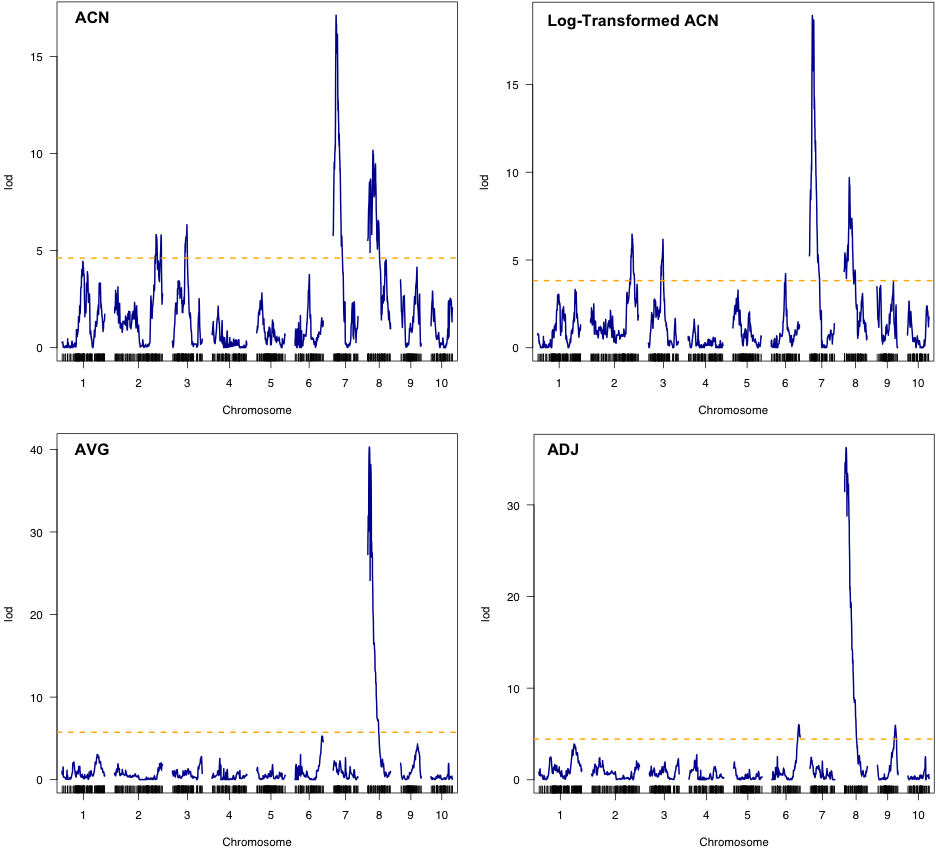


**Figure S2 (Cont.)**


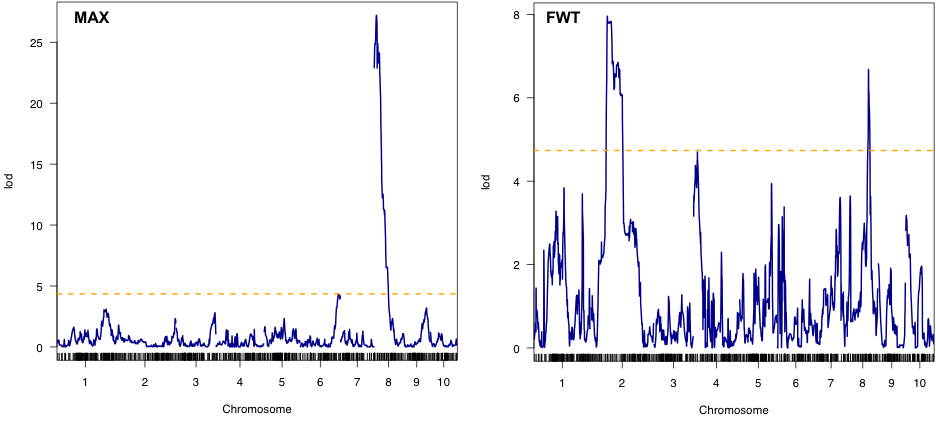


**Table S1:** Primers used in this study

| **Marker** | **Forward** | **Reverse** | **B73 RefGen v4 Position** | **Mo17 position** | **Annealing Temp (˚C)*** |
| --- | --- | --- | --- | --- | --- |
| **umc1974** | ACAAGGAGACCCTCCTCAGCTAGT | GTAAGCTGTGGCCATACTACCACC | 17,461,549 | 19,178,123 | 51 |
| **umc1913** | AAACAACTATCCATGTGGCTGACC | CGTTCAGTACAATTTGGCTCAGTG | 19,243,135 | 21,067,029 | 53 |
| **MPMAL1** | AATACTAACTGCCTACTGGTACTGT | GTGCAGCTCTCGTTAGAAAGAT | 19,585,194 | 21,311,272 | 52 |
| **MPMAL2** | AATTTCAGTGTGCCGTGCTA | CTTTACCTGCCTGCTTTGGAG | 20,367,292 | 21,935,151 | 52 |
| **MPMAL4** | ACATTTGCATGGCACTCACG | ACGTCGAGAGGTGCATAGAG | 21,787,204 | 23,412,424 | 53 |
| **umc1530** | GTGGCTCAACCTCTTCCTCCC | GGTTCATGGGGTAGACCAGCA | 22,931,854 | 24,429,960 | 53 |
| **umc1778** | GTGAACCATTGTAGCTGTCCCTG | GAGCTCGTACCTGTTCATGAGGAT | 24,528,278 | 25,951,420 | 57 |
| **umc2146** | GTCTCCGTCCACCTCCTGTG | GTCATGGGAATGTGCTGGATG | 25,814,989 | 27,095,521 | 55 |

*Using NEB Taq 5x Master Mix (cat no. m0285)
